# SBFI26 induces triple‐negative breast cancer cells ferroptosis via lipid peroxidation

**DOI:** 10.1111/jcmm.18212

**Published:** 2024-03-22

**Authors:** Gang He, Yiyuan Zhang, Yanjiao Feng, Tangcong Chen, Mei Liu, Yue Zeng, Xiaojing Yin, Shaokui Qu, Lifen Huang, Youqiang Ke, Li Liang, Jun Yan, Wei Liu

**Affiliations:** ^1^ Key Laboratory of Medicinal and Edible Plants Resources Development of Sichuan Education Department Sichuan Industrial Institute of Antibiotics, School of Pharmacy, Chengdu University Chengdu China

**Keywords:** ferroptosis, lipid peroxidation, SBFI26, TNBC, transcriptome

## Abstract

SBFI26, an inhibitor of FABP5, has been shown to suppress the proliferation and metastasis of tumour cells. However, the underlying mechanism by which SBFI26 induces ferroptosis in breast cancer cells remains largely unknown. Three breast cancer cell lines were treated with SBFI26 and CCK‐8 assessed cytotoxicity. Transcriptome was performed on the Illumina platform and verified by qPCR. Western blot evaluated protein levels. Malondialdehyde (MDA), total superoxide dismutase (T‐SOD), Fe, glutathione (GSH) and oxidized glutathione (GSSG) were measured. SBFI26 induced cell death time‐ and dose‐dependent, with a more significant inhibitory effect on MDA‐MB‐231 cells. Fer‐1, GSH and Vitamin C attenuated the effects but not erastin. RNA‐Seq analysis revealed that SBFI26 treatment significantly enriched differentially expressed genes related to ferroptosis. Furthermore, SBFI26 increased intracellular MDA, iron ion, and GSSG levels while decreasing T‐SOD, total glutathione (T‐GSH), and GSH levels.SBFI26 dose‐dependently up‐regulates the expression of HMOX1 and ALOX12 at both gene and protein levels, promoting ferroptosis. Similarly, it significantly increases the expression of SAT1, ALOX5, ALOX15, ALOXE3 and CHAC1 that, promoting ferroptosis while downregulating the NFE2L2 gene and protein that inhibit ferroptosis. SBFI26 leads to cellular accumulation of fatty acids, which triggers excess ferrous ions and subsequent lipid peroxidation for inducing ferroptosis.

## 1 INTRODUCTION

The latest cancer statistics indicate that breast cancer has surpassed lung cancer as the most lethal form worldwide, causing more female fatalities than any other type.^1^ Breast cancer is divided into several subtypes, including four molecular types: Luminal A, Luminal B, HER‐2 overexpressing breast cancer, and triple‐negative breast cancer (TNBC). About 15% ~ 20% of breast cancers are TNBC, which lacks the expression of human epidermal growth factor receptor (HER2), progesterone receptor (PR) and oestrogen receptor (ER), strong aggressiveness, easy recurrence and high cell viability.^2^, ^3^, ^4^ Current EGFR targeting and hormone therapy are ineffective for TNBC, so developing TNBC therapeutic drugs has been a hot topic in medical research.^5^, ^6^ Many targeted drugs have been studied, mainly divided into the following categories: PARP inhibitors, antiangiogenic drugs, EGFR inhibitors, AR antagonists, immune checkpoint inhibitors and antibody‐drug conjugates. However, the high‐frequency mutational nature of breast cancer drug targets makes these targeted drugs less effective in clinical treatment, so it is imperative to find new targets and develop more efficient and less toxic drugs.^7^, ^8^, ^9^

SBFI26 is classified as a cocaine monoester compound, and its core structure resembles the alkaloid found in *Incarvillea sinensis*, a Chinese herbal medicine.^10^ Early investigations have demonstrated that SBFI26 exhibits analgesic and anti‐inflammatory properties while concurrently functioning as a fatty acid transporter (FABP5) inhibitor.^11^ Fatty acid binding protein (FABP) exhibits a strong affinity for fatty acids and plays crucial roles in these molecules' transportation and metabolism.^12^, ^13^, ^14^ Numerous studies have demonstrated that FABP5 is highly expressed in malignant tumours,^15^, ^16^, ^17^ particularly in breast cancer, where its expression positively correlates with malignancy.^16^, ^18^ Therefore, targeting FABP5 may represent a promising therapeutic strategy for drug development. According to reports, SBFI26 demonstrates a strong binding affinity for FABP5/7 proteins.^19^ In vitro studies have demonstrated an inhibition rate exceeding 50%.^10^ In the eutectic complex of SBFI26 and FABP5, functional groups such as hydrogen bonds and salt bridges are formed to enhance the inhibitory activity of SBFI26.^19^ Studies have demonstrated that SBFI26 exhibits excellent therapeutic efficacy in mice with prostate cancer^20^, ^21^, ^22^ and exerts potent cytotoxicity against PC3M prostate cancer cells in vitro experiments.^10^, ^23^, ^24^ Furthermore, studies have shown that the down‐regulation of the FABP5 gene inhibits the function of the FABP5 protein in transporting fatty acids.^23^, ^25^

The primary mechanisms of action for antitumor compounds include apoptosis,^26^, ^27^, ^28^ pyroptosis,^29^, ^30^ necrotizing apoptosis,^31^, ^32^ and cell cycle arrest.^33^, ^34^ Recent research has also identified ferroptosis and cuproptosis as unique nonapoptotic forms of cell death characterized by intracellular iron accumulation,^35^ increased reactive oxygen species (ROS)^36^ and lipid peroxidation^37^ and imbalances between oxidative and antioxidant systems.^38^ Extensive studies have established that ferroptosis plays a critical part in cancer progression,^39^, ^40^ and triggering ferroptosis could be a viable approach for anticancer treatments and mitigating chemotherapy resistance in cancerous cells.^41^, ^42^, ^43^, ^44^, ^45^, ^46^ Previous research has demonstrated that SBFI26 elicits intracellular apoptotic signals and induces apoptosis in PC3M cells by regulating the FABP5‐VEGF‐PPAR axis.^10^, ^23^ While previous studies have shown that SBFI26 can impede the proliferation of TNBC cells, its inhibitory effects on breast cancer have not yet been reported.^47^

In this study, we investigated the impact of SBFI26 on breast cancer cell proliferation and analysed the transcriptome data following treatment with SBFI26 in TNBC cells. Through KEGG and GO analysis of transcriptomic data, it was observed that SBFI26 treatment up‐regulated the expression of ferroptosis‐related genes in TNBC cells compared to the control group. This finding suggests a strong correlation between SBFI26‐induced cell death and ferroptosis.

Moreover, DEGs analysis showed that differential expression of genes is regulated by those involved in the ferroptosis molecular pathway, which is driven by iron metabolism, lipid peroxidation and redox system imbalance. Therefore, we conducted a study on the molecular pathway of SBFI26 to induce ferroptosis in TNBC, as it has significant potential as a small‐molecule compound for intervening in the oncogenic process through the mechanism of ferroptosis.

## 2 MATERIALS AND METHODS

### 2.1 Reagents and antibodies

Reagents: ferrostatin‐1 (CAS:347174–05‐4) and erastin (CAS:571203–78‐6) were all purchased from MedChemExpress (shanghai; China). SBFI26 (CAS:1541207–06‐0) was purchased from GLPBIO Technology (American). Antibodies: Primary antibodies were used as following: anti‐β‐actin antibody (1:1000, mAbcam 8226, Abcam), anti‐ALOX5 (1:500–1:1000, R1512‐14, HuaBio), anti‐ALOX12 (1:2000, AP8877B Abcepta Biotech), anti‐NFE2L2 (1:1000–1:2000, R1312‐8, HuaBio), anti‐GPX4 (1:500–1:2000, ET1706‐45, HuaBio), anti‐HO‐1 (1:1000, ET1604‐45, HuaBio), anti‐ATF4 (1:500–1:2000, ET1612‐37, HuaBio), secondary antibody was purchased from Beyotime (Shanghai, China).

### 2.2 Cell lines and culture conditions

The MCF‐7, MDA‐MB‐468, and MDA‐MB‐231 cell lines derived from human breast cancer were acquired from ATCC and cultured in a 37°C incubator with 5% CO_2_ under humid conditions; MDA‐MB‐468 cells were cultured using RPMI‐1640 medium, and MDA‐MB‐231 and MCF‐7 cells were cultured using Dulbecco's modified Eagle's medium (Gibco, Thermo Fisher Scientific, American), contain with 10% fetal bovine serum (Gibco, Thermo Fisher Scientific, American), 1% antibiotics (penicillin 10000 U/mL, streptomycin 100 mg/mL) (Solarbio, Beijing, China), respectively.

### 2.3 Cell viability assay

Cells suspension (5 × 10^4^, 100 μL) were seeded in 96‐well plates, and six replicates were included in each group for overnight adhesion culture at 37°C with 5% CO_2_. The control group was treated with DMSO, while the SBFI26 treatment groups received concentrations of 50, 75, 100, 125 and 150 μM at 37°C for 12, 24 and 48 h respectively. Cytotoxicity of SBFI26 was evaluated using the CCK‐8 assay (ZOMANBIO). After removing the medium and adding fresh medium containing CCK‐8 reagent (10 microliters/well), absorbance detection was performed at a wavelength of 450 nm using a microplate reader (BioTek). The calculation of cell viability was performed using the formula specified in the CCK‐8 manual.

### 2.4 Cells processing, RNA isolation and library preparation

The cells were seeded in 90‐mm dishes, cultured overnight and subsequently treated with 100 μM SBFI26 for 24 h. The control group did not undergo SBFI26 treatment; each consisted of three replicates. After a culture period of 24 h, the cells were collected and preserved by adding a 1 mL TRIzol reagent prior to RNA extraction and sequencing.^48^, ^49^

The manufacturer's protocol used the TRIzol reagent to extract the total RNA. The NanoDrop 2000 spectrophotometer (Thermo Scientific, USA) was employed to evaluate RNA purity and quantification. Additionally, RNA integrity was assessed using Bioanalyzer (Agilent Technologies, Santa Clara, CA, USA). Libraries were constructed on Illumina platforms. Finally, OE Biotech Co., Ltd. (Shanghai, China) performed transcriptome sequencing and analysis.

### 2.5 Iron assay

Alterations in iron levels serve as a pivotal biomarker for identifying ferroptosis. Intracellular total iron and ferrous ion content were measured using the iron assay kits (E‐BC‐K880‐M and E‐BC‐K881‐M, Elabscience), respectively. Briefly, cells were collected with a cell scraper and 0.2 mL of buffer lysate was added to approximately 1 × 10^6^ cells per sample. The mixture was evenly mixed, incubated on ice for 10 min, centrifuged at 15000 × g for 10 min, and the supernatant was used for measurement following kit instructions. Total iron samples were incubated at 37°C for 40 min, while ferrous content samples were incubated at 37°C for 10 min before measuring absorbance at 593 nm using an enzyme‐labelled. Calculate the total iron and ferrous content according to the instructions.

### 2.6 Lipid peroxidation assay

Lipid peroxidation levels were evaluated by measuring intracellular Malondialdehyde (MDA) content using an enzyme‐linked method based on the degree of reaction between MDA and TBA. In brief, approximately 3 × 10^6^ cells were collected from each group, separated with a cell scraper and washed three times with PBS to obtain cell precipitates. The extract was added according to kit instructions, followed by ultrasound‐assisted fragmentation at 90 W for 4 s/time with a 2 s gap for 10 min. The protein concentration of the resulting cell fragmentation fluid was determined using the BCA kit (E‐BC‐K318‐M, Elabscience). After water bath treatment at 100°C for 40 min and cooling to room temperature, samples were centrifuged at 1078 × g for 10 min before taking out supernatant (0.25 mL) into a microplate reader at a wavelength of 532 nm to determine the absorbance value and calculate MDA content as instructed in MDA Colorimetric Assay Kit (E‐BC‐K028‐M, Elabscience).

### 2.7 Detection of total superoxide dismutase (T‐SOD) assay

A T‐SOD activity assay kit (Hydroxylamine Method) purchased from the Nanjing Institute of Bioengineering, China, was utilized to examine the intracellular T‐SOD activity. Briefly, cells (10^6^) were homogenized in 500 μL PBS (0.01 M, pH 7.4). After homogenization, the supernatant was collected by centrifugation at 10,000 × g for 10 min at 4°C and kept on ice for measurement. The protein content of the sample was determined using a BCA kit, and T‐SOD activity was assessed following the provided instructions. The absorbance value of the reaction solution was measured at 550 nm. Enzyme activity was defined as one SOD unit (U) when the SOD inhibition rate reached 50% per mg of protein in a 1 mL reaction solution volume.

### 2.8 Glutathione content assay

Glutathione (GSH) is a crucial barrier against cell ferroptosis; the total glutathione (T‐GSH), oxidized glutathione (GSSG) and GSH were measured using T‐GSH and GSSG colourimetric assay kit (E‐BC‐K097‐M, Elabscience).In brief, the cells from each treatment group were collected and lysed with a ratio of 400 μL lysis solution (reaction solution 3) per 10^6^ cells, followed by mechanical homogenization to ensure complete cell disruption (no visible cell precipitation was observed under the microscope). The resulting mixture was then centrifuged at 10000 × g at 4°C for 10 min, and the supernatant was collected and kept on ice for further analysis. T‐GSH content was determined using a kit method, where absorbance values of each well were measured at 412 nm using an enzyme‐labelled instrument. GSSG content was subsequently determined by removing GSH before conducting a sample reaction and measuring absorbance values again at 412 nm with a microplate reader. Finally, GSH content was calculated as T‐GSH minus GSSG.

### 2.9 Ferroptosis inhibitors and promoters on cell proliferation combined by SBFI26 treatment

Cells (5 × 10^4^) were cultured in 96‐well plates and adhered overnight. Subsequently, the cells were treated with various combinations of reagents as follows: (1) SBFI26 (0, 50, 100, 150 μM) in combination with Fer‐1 (60 nM), (2) erastin (0, 1, 2, 3, 4, 5 μM), (3) erastin (0, 2, 3, 4, 5 μM) in combination with Fer‐1 (60 nM),^50^, ^51^ (4) SBFI26 (100 μM) combined with erastin (3 μM)^50^, ^52^, ^53^ and Fer‐1 (60 nM), (5) SBFI26 (100 μM) combined with GSH (0,150,250 mg/mL), and finally, (6) SBFI26 (100 μM) combined with VitaminC (0, 150, 250 μM). Cell viability was assessed using a CCK‐8 assay (ZOMANBIO).

### 2.10 Quantitative real‐time polymerase chain reaction (qPCR) assay

Total RNA was extracted with the Cell RNA Rapid Extraction Kit (ZOMANBIO, China) and reversed to cDNA using a reverse transcription kit (containing thermosensitive double‐stranded DNase) (Biosharp, China) 0.2 × HQ SYBR qPCR Mix (High ROX) (ZOMANBIO, China) for primer validation and amplification of the gene of interest, performing reaction conditions in predenaturation at 95°C for 30s and 40 cycles in denaturation 95°C 10s and annealing extension 60°C 30 s, GAPDH gene was used as internal reference gene, Quantitative results were analysed using the 2^−ΔΔCt^ method, and all primers in this experiment were presented in the supporting material (Table 1).

**TABLE 1:** Primers used for Q‐PCR analysis.

Gene	Forward primer 5′ → 3′	Reverse primer 5′ → 3′
GAPDH	5'‐ATCAATGGAAATCCCATCACCA‐3'	5'‐GACTCCACGACGTACTCAGCG‐3'
ATF4	5'‐TCAAACCTCATGGGTTCTCC‐3'	5'‐GTGTCATCCAACGTGGTCAG‐3'
HO‐1	5'‐TTCAGCATCCTCAGTTCC‐3'	5'‐CCGTGTCAACAAGGATAC‐3'
NFE2L2	5'‐TCCAGTCAGAAACCAGTGGAT‐3'	5'‐GAATGTCTGCGCCAAAAGCTG‐3'
ATF3	5'‐AAGAACGAGAAGCAGCATTTGAT‐3'	5'‐TTCTGAGCCCGGACAATACAC‐3'
CHAC1	5'‐CCTGAAGTACCTGAATGTGCGAGA‐3'	5'‐GCAGCAAGTATTCAAGGTTGTGGC‐3'
GPX4	5'‐TTCCCGTGTAACCAGTTCG‐3'	5'‐CGGCGAACTCTTTGATCTCT‐3'
SAT1	5'‐CCGTGGATTGGCAAGTTATT‐3'	5'‐TCCAACCCTCTTCACTGGAC‐3'
ALOX5	5'‐CCTCAGGCTTCCCCAAGT‐3'	5'‐GAAGATCACCACGGTCAGGT‐3'
ALOX12	5'‐GCTCCTGGAACTGCCTAGAA‐3'	5'‐TCATCATCCTGCCAGCACT‐3'
ALOX15	5'‐AGCCTGATGGGAAACTCTTG‐3'	5'‐AGGTGGTGGGGATCCTGT‐3'
ALOXE3	5'‐TGTATTTCGCTTTCCTGACC‐3'	5'‐CTTGTTTGCTTGCCTCTGA‐3'
TP53	5'‐ACAGCTTTGAGGTGCGTGTTT‐3'	5'‐CCCTTTCTTGCGGAGATTCTCT‐3'

### 2.11 Western blot analysis

Protein was extracted using RIPA cell lysate buffer containing protease inhibitors and phosphatase inhibitors, and the total protein concentration was determined by the BCA Kit, and the protein concentration of cells at different administered concentrations was relatively quantified at a standard of 1 μg/μl before loading. After 12% SDS PAGE gel electrophoresis, the total protein was separated and transferred to a nitrocellulose membrane (Immobilon TM‐P; Millipore, United States) (Piscataway, New Jersey). After 1 h of blockade with 5% skim milk powder, after washing, incubate the specific primary antibody overnight at 4°C, then room temperature co‐incubation of blots with HRP‐labelled IgG (A0208, A0216; Beyotime) secondary antibody 1 h.

### 2.12 GEPIA dataset analysis

GEPIA dataset is a newly developed interactive web server for analysing RNA sequencing expression data from 9736 tumours and 8587 standard samples from the Cancer Genome Atlas and Genotype‐Tissue Expression (GTEx) project, using a standard processing pipeline.^54^ GEPIA provides customizable features such as tumour/normal differential expression analysis, cancer type or pathological stage analysis, patient survival analysis, similarity gene detection, correlation analysis and dimensionality reduction analysis, and prognostic signal verification based on optimal cut‐off values.

### 2.13 Kaplan–Meier plotter

The association between the expression of key genes associated with ferroptosis in breast tumour samples and patient survival to discover and validate survival‐related biomarkers were evaluated using the online database Kaplan–Meier Plotter^55^ (www.kmplot.com), which contains gene expression data and survival information (http://kmplot.com/analysis/index.php?p=service&cancer=breast) for breast cancer patients. To analyse the OS patient sample of breast cancer patients divided into two groups by median expression (high expression versus low expression) and assessed by Kaplan–Meier survival plot, the hazard ratio (HR) had 95% confidence intervals (CIs) and logarithmic ranking p‐values. The set of probes associated with ferroptosis is selected to obtain a Kaplan–Meier plot where risk numbers are below the main plot.

## 3 RESULTS

### 3.1 SBFI26 suppresses breast cancer cell growth and induces cell death

**FIGURE 1:**
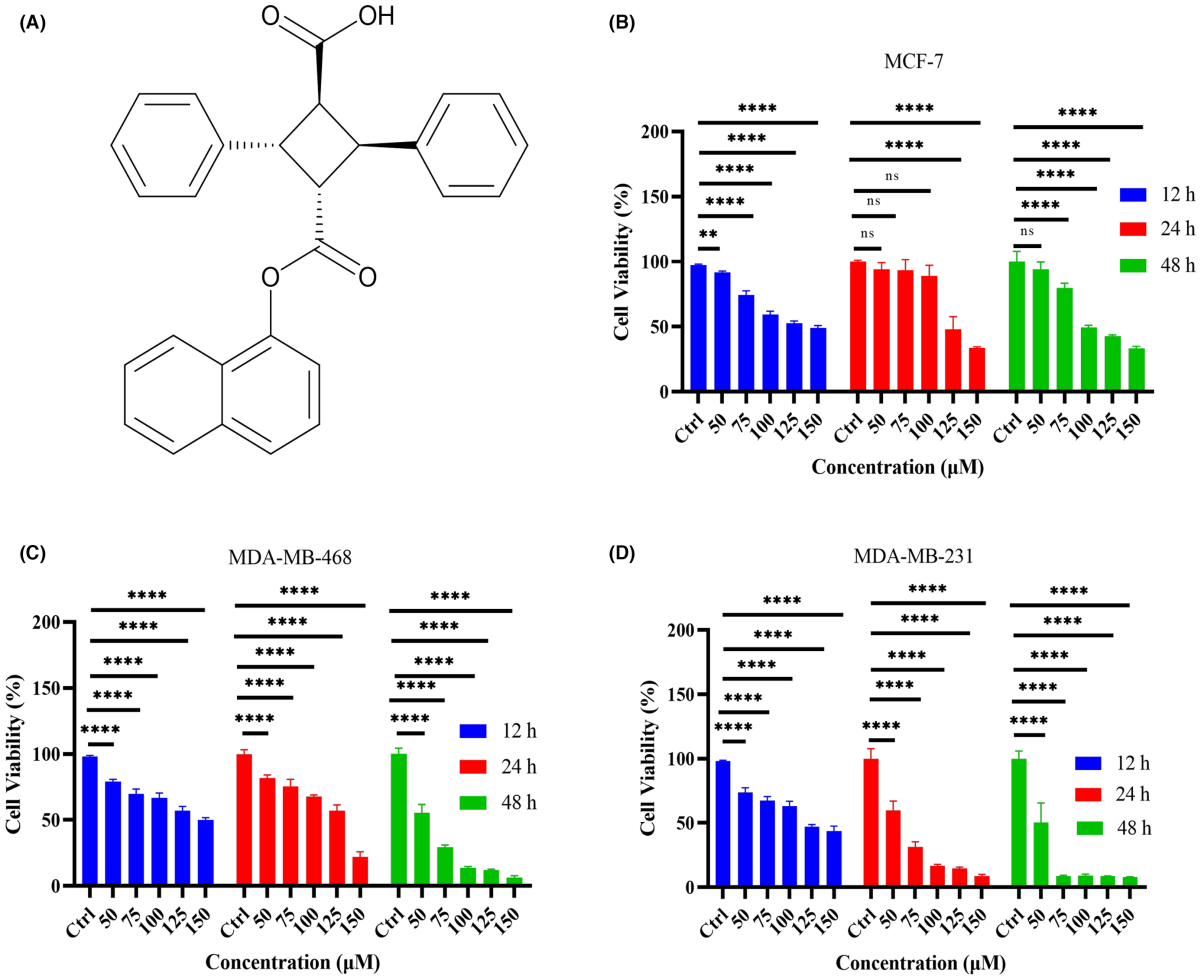
SBFI26 suppresses cell growth. (A) The chemical structure of SBFI26. (B–D) Cell viability of MCF‐7, MDA‐MB‐468, and MDA‐MB‐231 cells were measured by CCK8 assay after treatment with indicate concentration of SBFI26 (50, 75, 100,125,150 μM) at 12, 24, 48 h. Statistical analysis was carried out between the SBFI26‐treated group and the untreated group. Data were presented as Mean ± SD. Data were analysed using one‐way ANOVA, with* *p* < 0.05, ** *p* < 0.01, *** *p* < 0.001, **** *p* < 0.0001; ns, not significant.

The chemical structure of SBFI26 is depicted in Figure 1A. To evaluate the cytotoxicity of SBFI26 on breast cancer cell lines, MCF‐7 (Figure 1B), MDA‐MB‐468(Figure 1C) and MDA‐MB‐231 (Figure 1D) cells were treated with varying concentrations of SBFI26 for different durations (12, 24, or 48 h). Cell viability was evaluated by performing a CCK‐8 kit assay. As depicted in Figures 1B–D, The result demonstrates that SBFI26 significantly reduced the proliferation of MDA‐MB‐468 and MDA‐MB‐231 cell lines in a concentration‐dependent and time‐dependent manner. The inhibitory effects of SBFI26 on cell proliferation significantly differed from those of the control group after 12 h of treatment across all three cell lines. After 24 h of treatment, only MDA‐MB‐468 and MDA‐MB‐231 cells showed significant differences in inhibition compared to the control group at high doses (125 and 150 μM). After 48 h of treatment, SBFI26 exhibited significant inhibitory activity on MDA‐MB‐468 and MDA‐MB‐231 cells compared to the control group. However, MCF‐7 has no significant time dependence, the inhibition of SBFI26 on MCF‐7 cells was observed only at doses greater than 100 μM and the inhibition rate could reach 50%. Notably, SBFI‐26 demonstrated superior efficacy against MDA‐MB‐231 cells, which belong to the TNBC subtype and exhibit high expression levels of FABP5 protein that renders them more sensitive to SBFI26. Therefore, subsequent studies were conducted using the MDA‐MB‐231 cell line.

### 3.2 Transcriptome analysis and DEG identification

**FIGURE 2:**
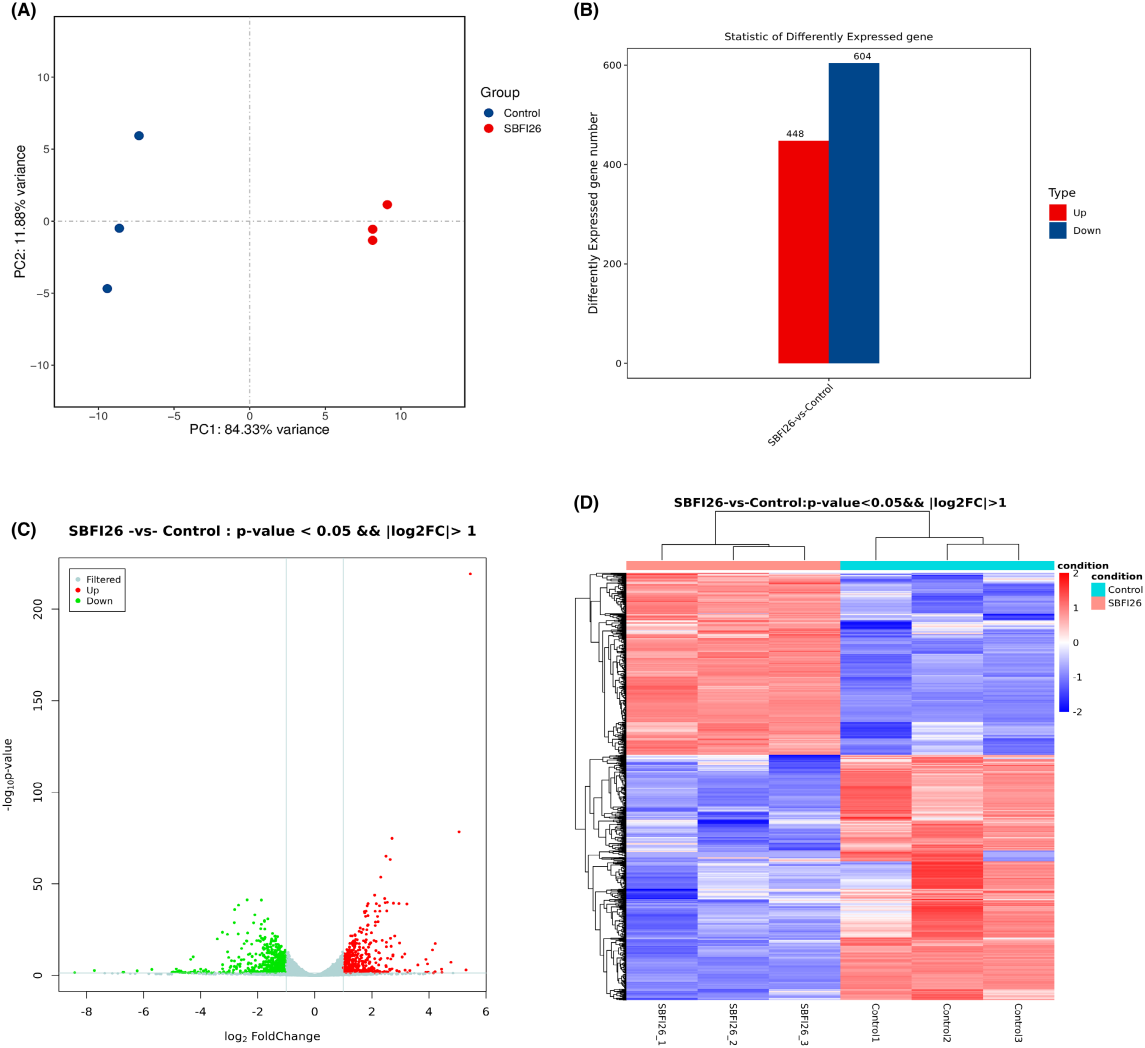
The transcriptome is identified as the principal component and analysis of Ctrl, SBFI26‐treated differential genes. (A) Principal components analysis (PCA) plot. (B) Statistical histogram of differentially expressed genes. (C) Volcano map of differential expression genes, the horizontal axis is log_2_FoldChange, and the vertical axis is‐log_10_ q‐value. (D) Cluster map of differentially expressed genes.

PCA analysis was conducted to provide an overview of the transcriptomic variations. As depicted in Figure 2A, based on PC1 (84.33%) and PC2 (11.88%), the samples were distinctly separated into two groups, indicating significant differences in transcriptomic profiles between the SBFI26 treatment and control groups. The greatest number of genes exhibiting differential expression (DEGs) was observed after 24 h, with 448 genes showing up‐regulation and 604 genes displaying down‐regulation. (Figure 2B). Volcano plots of DEGs are shown in Figure 2C; Grey is the gene with a non‐significant difference, and red and green are the genes with a significant difference. The hierarchical clustering analysis (HCA) of differential gene expression levels (Figure 2D) revealed that the SBFI26 treatment groups formed a distinct cluster, consistent with the results obtained from the PCA analysis.

### 3.3 GO analysis of DEGs

**FIGURE 3:**
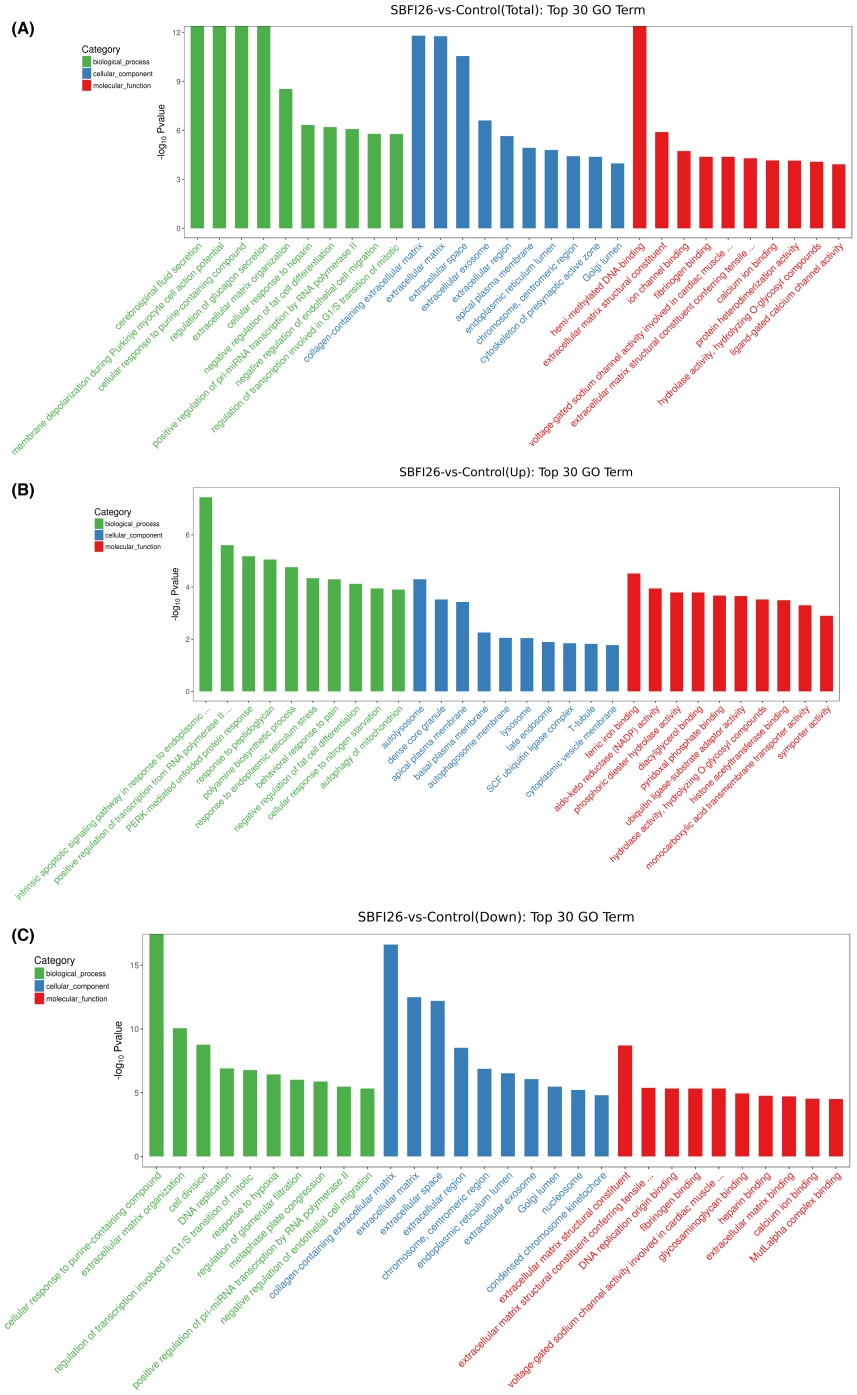
Top 20 GO terms for DEGs between the SBFI26 and Control groups. (A) TOP20 GO Term (total). (B) TOP20 GO Term (UP). (C) TOP20 GO Term (down).

GO enrichment analysis can be categorized into three levels, with Level 1 consisting of three GO items: biological process (BP), cellular component (CC) and molecular function (MF). Level 2 includes 64 GO items, such as biological adhesion, cell and binding, while Level 3 encompasses tens of thousands of entries used for regular enrichment. The top 30 GO terms for DEGs between the SBFI26 and Control groups are presented in Figure 3A. The results of the GO enrichment analysis revealed that the DEGs were primarily involved in various BP, including cerebrospinal fluid secretion, membrane depolarization during Purkinje myocyte cell action potential, cellular response to purine‐containing compounds and regulation of glucagon secretion. Additionally, these DEGs were found to be associated with specific cell components such as collagen‐containing extracellular matrix, extracellular matrix, extracellular space and exosomes. Furthermore, they exhibited MF such as hemimethylated DNA binding, extracellular matrix structural constituent and ion channel binding.

As shown in Figure 3B,C Top 30 GO Term results showed that up‐regulated BP included (such as intrinsic apoptotic signalling pathway in response to endoplasmic reticulum stress, enhancement of transcriptional activity from RNA polymerase II promoter in reaction to endoplasmic reticulum stress), down‐regulated BP included (such as autolysosome, dense core granule); up‐regulated CC included (such as ferric iron binding, aldo‐keto reductase (NADP) activity), down‐regulated CC included(such as cellular response to purine‐containing compound, extracellular matrix organization), up‐regulated MFs included (such as collagen‐containing extracellular matrix, extracellular matrix), down‐regulated MFs included (such as extracellular matrix structural constituent, extracellular matrix structural constituent conferring tensile strength). The distribution of differential genes and all genes at GO Level2 and the distribution of up‐regulated and down‐regulated differential genes at GO Level2 were compared and presented in Figure S1.

### 3.4 KEGG pathway analysis of DEGs

**FIGURE 4:**
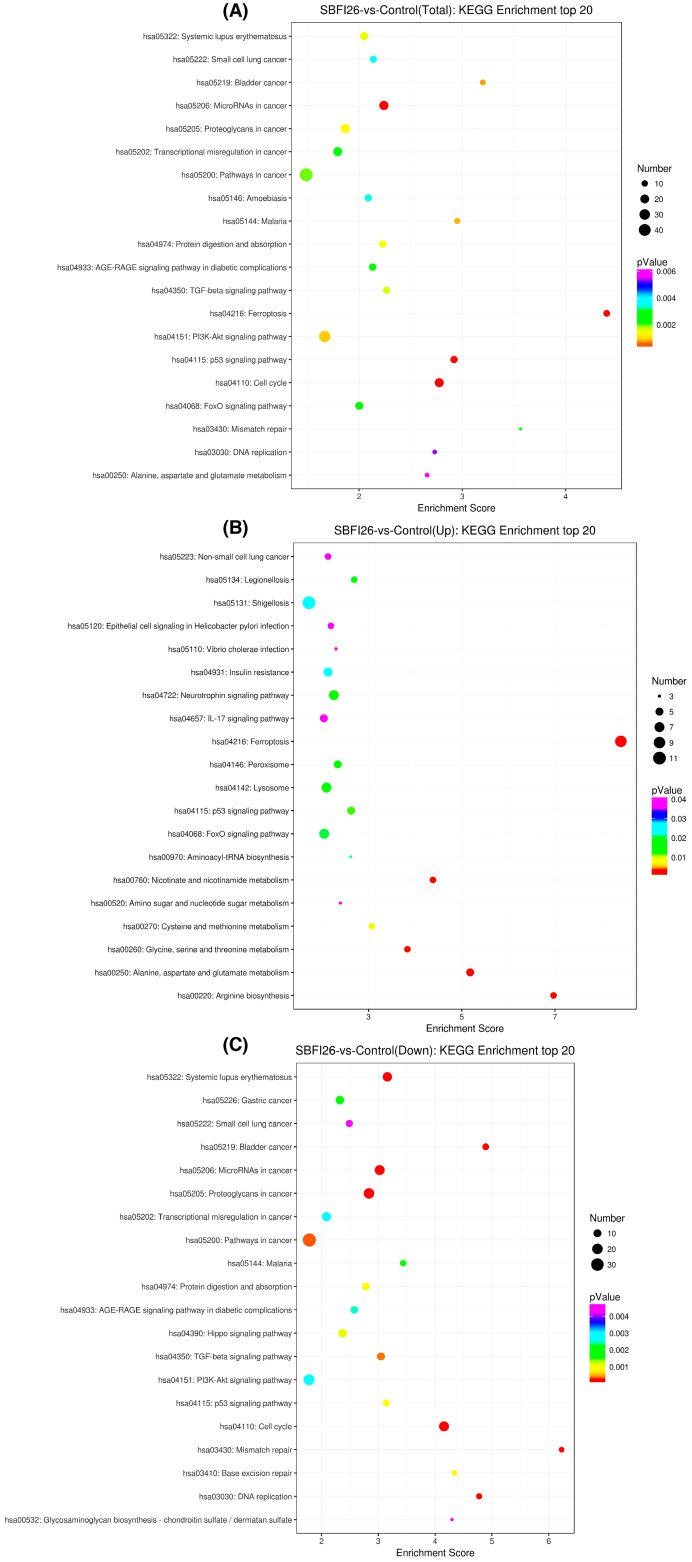
Top 20 KEGG enrichment for DEGs between the SBFI26 and Control groups. (A) Top 20 KEGG enrichment (total). (B) Top 20 KEGG enrichment (UP). (C) Top 20 KEGG enrichment (down). The horizontal axis enrichment score in the figure is the enrichment score; the more significant the bubble entry contains, the greater number of differential protein‐coding genes, the bubble colour changes from purple‐blue‐green‐red, and the smaller the enrichment *p*‐value value, the greater the significance.

Based on the KEGG database, the pathways were annotated for differentially expressed genes (DEGs) at three levels. Level 1 encompasses six major categories: Metabolism, Genetic Information Processing, Environmental Information Processing, Cellular Processes, Organismal Systems and Human Diseases (specific species annotations may be censored). Level 2 comprises 44 subcategories, including Cell growth and death, Transcription and Development. Finally, level 3 encompasses hundreds of pathways. All enriched pathways of DEGs in the SBFI26 and Control groups are shown in Figure 4.

As depicted in Figure 4A, the KEGG Enrichment analysis of the top 20 (Total) revealed that the DEGs were predominantly associated with ferroptosis, microRNA in cancer, malaria, cell cycle and p53‐mediated signalling pathway. Notably, the ferroptosis pathway exhibited the highest enrichment score and the lowest P value among all pathways analysed. As shown in Figure 4B, the top 20 DGEs with significantly up‐regulated KEGG enrichment were primarily involved in Ferroptosis, Alanine, Aspartate and Glutamate Metabolism, Nicotinate and Nicotinamide Metabolism. Ferroptosis exhibited the highest enrichment score with the lowest P value and nine DEGs identified. The top 20 DGEs with significantly down‐regulated KEGG enrichment are shown in Figure 4C, where the pathways with high enrichment scores and smallest p‐values were mainly involved in such mismatch repair and cell cycle. The distribution of differential genes and all genes at KEGG Level2 and the distribution of up‐regulated and down‐regulated differential genes at KEGG Level2 were compared and presented in Figure S2. The KEGG analysis of DEGs revealed that SBFI26 could trigger cell death via ferroptosis.

### 3.5 Effect of ferroptosis inhibitors and inducers combined with SBFI26 treatment on MDA‐MB‐231 cell proliferation

**FIGURE 5:**
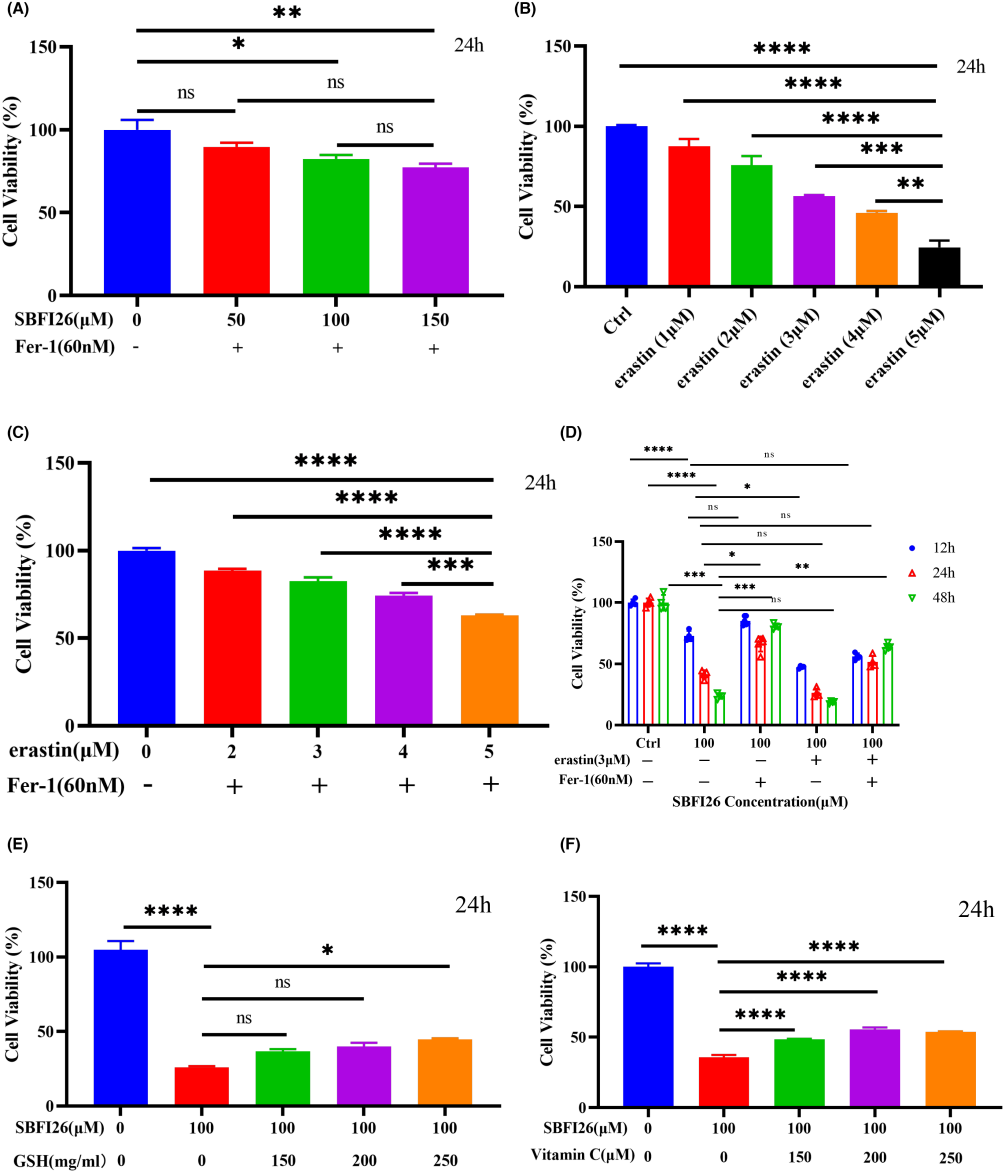
SBFI26 in combination with Fer‐1, erastin, GSH, and Vitamin C on cell viability. (A) SBFI26 (50,100,150 μM) combined with Fer‐1(60 nM) on MDA‐MB‐231 cell viability. (B) Erastin (1, 2, 3, 4, 5 μM) was treated for 24 h on MDA‐MB‐231 cell viability. (C) Erastin (2, 3, 4, 5 μM) combined with Fer‐1 (60 nM) on MDA‐MB‐231 cell viability. (D) SBFI26 (100 μM) combined with or without Fer‐1(60 nM) and erastin (3 μM) treated for 12, 24, 48 h on MDA‐MB‐231 cell viability. (E) SBFI26 (100 μM) combined with GSH (150,200,250 mg/mL) treated for 24 h on MDA‐MB‐231 cell viability. (F) SBFI26 (100 μM) combined with VitaminC (150,200,250 μM) treated for 24 h on MDA‐MB‐231 cell viability. Data were presented as Mean ± SD. Data were analysed using one‐way ANOVA, with* *p* < 0.05, ** *p* < 0.01, *** *p* < 0.001, **** *p* < 0.0001; ns, not significant.

As depicted in Figures 1D and 5A, treatment of MDA‐MB‐231 cells with SBFI26 alone at concentrations of 50 μM, 100 μM and 150 μM for a duration of 24 h significantly reduced cell viability compared to the control group (*p* < 0.0001). However, co‐treatment with Fer‐1 (60 nM) for the same time period improved cell viability compared to the control group. Notably, there was no significant difference between the 50 μM dose group and the control group; however, significant differences were observed between the 100 μM and 150 μM groups (*p* < 0.05, *p* < 0.01). The ferroptosis inhibitor (Fer‐1) effectively prevented SBFI26‐induced cell death in MDA‐MB‐231 cells. Erastin, a type I ferroptosis inducer, significantly induces cell death, as demonstrated in Figure 5B. After treatment with Erastin (5 μM) for 24 h, the cell viability of cells was 24.61% (*p* < 0.0001). As shown in Figure 5C, treatment with Fer‐1 at a concentration of 60 nM significantly increased the cell viability to 70% compared with the 24.61% cell viability in the Erastin (5 μM) group (*p* < 0.0001). As depicted in Figure 5D, the cell viability was increased by 1.16‐folds after 12 h of treatment with SBFI26 (100 μM) combined with Fer‐1 (60 nm), compared to the group treated with SBFI26 (100 μM) alone. Conversely, the cell viability decreased by 0.56‐fold after 12 h of treatment with SBFI26 (100 μM) combined with Erastin (3 μM). The combination of SBFI26 (100 μM), Fer‐1(60 nm), and Erastin(3 μM) resulted in a decrease in cell viability by 0.77‐fold after 12 h of treatment, while an increase by 1.25‐fold was observed after 24 h of treatment compared to that treated only with SBFI26 (100 μM). After treating for a duration of up to 48 h with SBFI26 (100 μM) combined with Fer‐1 (60 nm), the cell viability increased by 3.41‐folds compared to that of SBFI26 (100 μM) alone. Treatment with SBFI26 (100 μM) in combination with Erastin (3 μM) decreased significantly by 0.79‐folds after 48 h of treatment, but there was an increase of 2.69‐folds after 48 h of treatment when combined with Fer‐1 (60 nm) and Erastin (3 μM). The results demonstrate that SBFI26 induces ferroptosis in MDA‐MB‐231 cells, with Fer‐1 inhibiting SBFI26‐induced ferroptosis and Erastin synergizing it.

Ferroptosis's most distinctive biological feature is the intracellular lipid peroxidation‐induced damage to biomembranes, necessitating antioxidant supplementation for balancing intracellular peroxidation levels. As shown in Figures 5E,F, compared with the control group, the addition of GSH (*p* < 0.05) and Vitamin C (*p* < 0.0001) increased the concentration of antioxidants, and the cell viability of cells was also significantly improved. Specifically, when compared with the SBFI26 (100 μM) treatment group, added GSH (100 mg/mL) exhibited a remarkable 1.8‐fold increase in cell viability, while those treated with Vitamin C (100 μM) showed a 1.4‐fold improvement.

### 3.6 Determination of MDA, T‐SOD, GSH and iron in MDA‐MB‐231 cell

**FIGURE 6:**
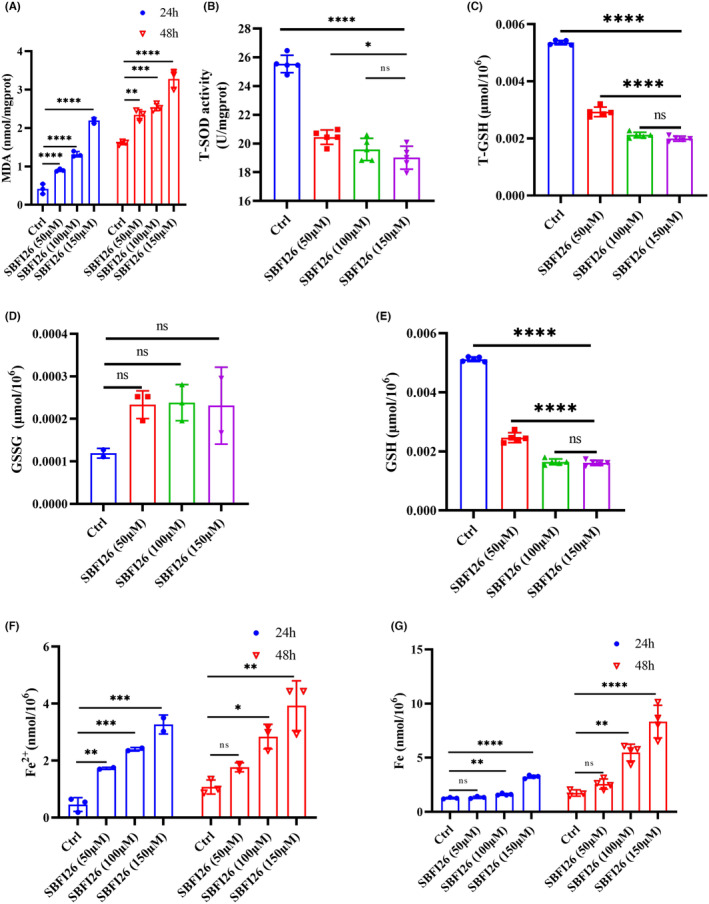
SBFI26 increases intracellular levels of ferrous, total iron, MDA, and oxidized glutathione, reduces T‐SOD activity, and total glutathione and reduced glutathione in MDA‐MB‐231 cells. (A) Quantification of cellular MDA levels using the TBA method at 24, 48 h. (B) The level of T‐SOD in cells treated with SBFI26 (50, 100, 150 μM) at 24 h. (C)Total intracellular glutathione, (D) Oxidized glutathione, and (E) Reduced glutathione determined using DTNB reagent treated with SBFI26 (50, 100, 150 μM) at 24 h. (F) Intracellular ferrous ions and (G) Total iron levels treated with SBFI26 (50, 100, 150 μM) at 24,48 h. Data were presented as Mean ± SD. Data were analysed using one‐way ANOVA, with* *p* < 0.05, ** *p* < 0.01, *** *p* < 0.001, **** *p* < 0.0001; ns, not significant.

An iron‐dependent accumulation of lipid peroxides characterizes ferroptosis well. As depicted in Figure 6A, MDA is a by‐product of lipid peroxidation. The MDA content of the treatment group with SBFI26 for 24 and 48 h was significantly more dose‐dependent compared to the control group. Specifically, treatment with 150 μM SBFI26 resulted in a 5.29‐fold increase in MDA content in the 24‐h group and a 2.03‐fold increase in the 48‐h group compared to the control group. SOD, GSH/GSSG, and Fe^2+^/Fe played a role in ferroptosis. After 24 h of treatment with SBFI26, T‐SOD content significantly reduced with the increase of SBFI26 concentration compared to the control group (*p* < 0.0001); The content of the SBFI26 (100 μM) treatment group was 0.74‐fold lower than that in the control group(Figure 6B). The contents of T‐GSH and GSH decreased significantly with the increase of SBFI26 concentration compared with the control group (*p* < 0.001), and GSSG content showed an increasing trend (*p* > 0.05). The levels of T‐GSH and GSH in the SBFI26 (100 μM) treatment group were 0.39‐fold and 0.31‐fold lower than those in the control group, respectively (Figure 6C–E). Compared to the control group, treatment with SBFI26 (100 μM) for 24 and 48 h resulted in a concentration‐dependent increase in levels of Fe^2+^ and total iron (*p* < 0.001). Following 24 h of treatment, the SBFI26 (100 μM) group exhibited a significant elevation of Fe^2+^ content by 7.1‐fold and total iron content by 2.55‐fold compared to the control group (Figure 6F,G).

### 3.7 In Vitro validation of genes and protein ferroptosis

**FIGURE 7:**
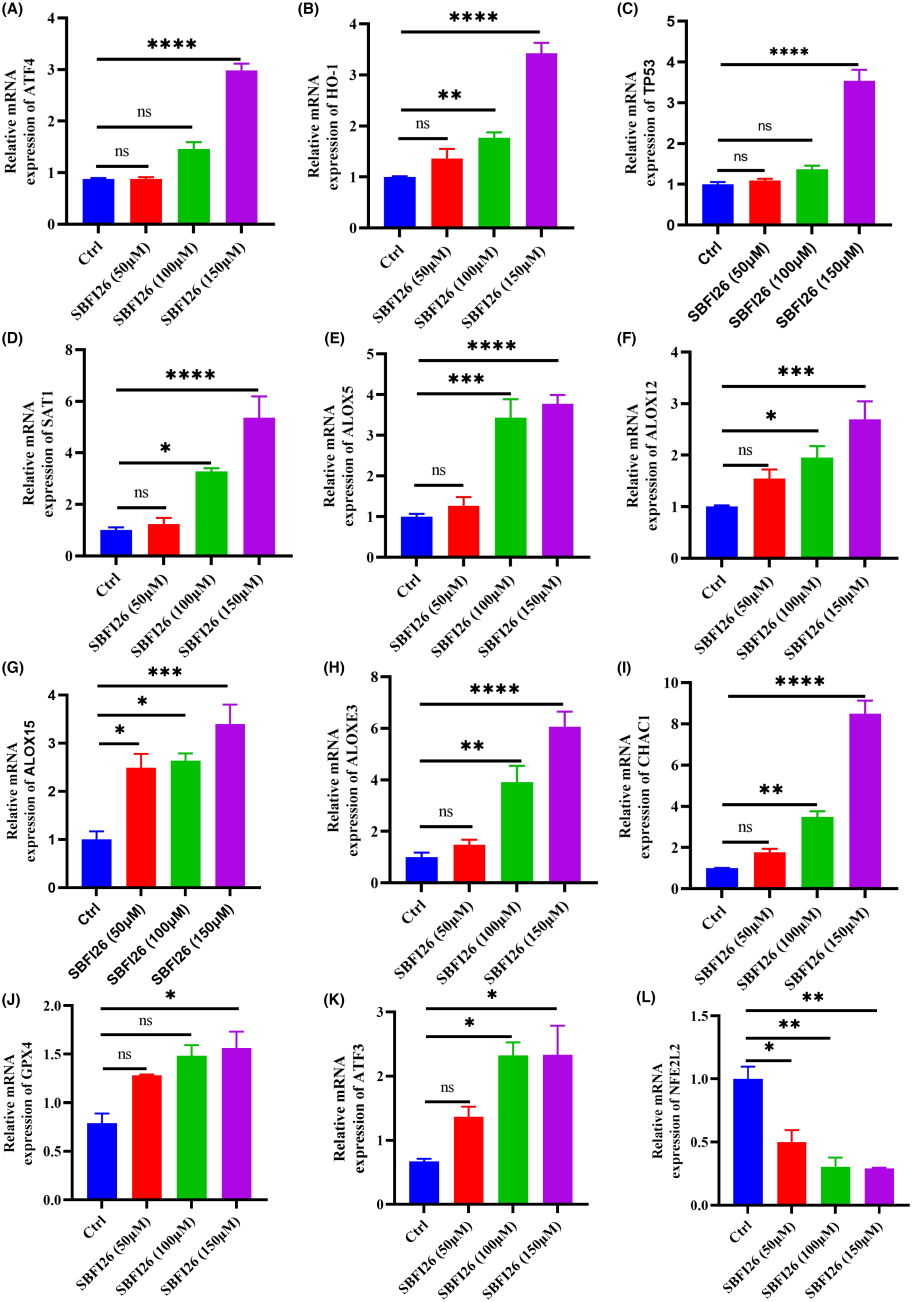
The relative mRNA levels of genes following treatment with SBFI26 compared with the untreated group, GAPDH gene as an internal reference. ATF4 (A), HO‐1 (B), TP53 (C), SAT1 (D), ALOX5 (E), ALOX12 (F), ALOX15 (G), ALOXE3 (H), CHAC1 (I), GPX4 (J), ATF3 (K), NFE2L2 (L). Data were presented as Mean ± SD. Data were analysed using one‐way ANOVA, with* *p* < 0.05, ** *p* < 0.01, *** *p* < 0.001, **** *p* < 0.0001; ns, not significant.

**FIGURE 8:**
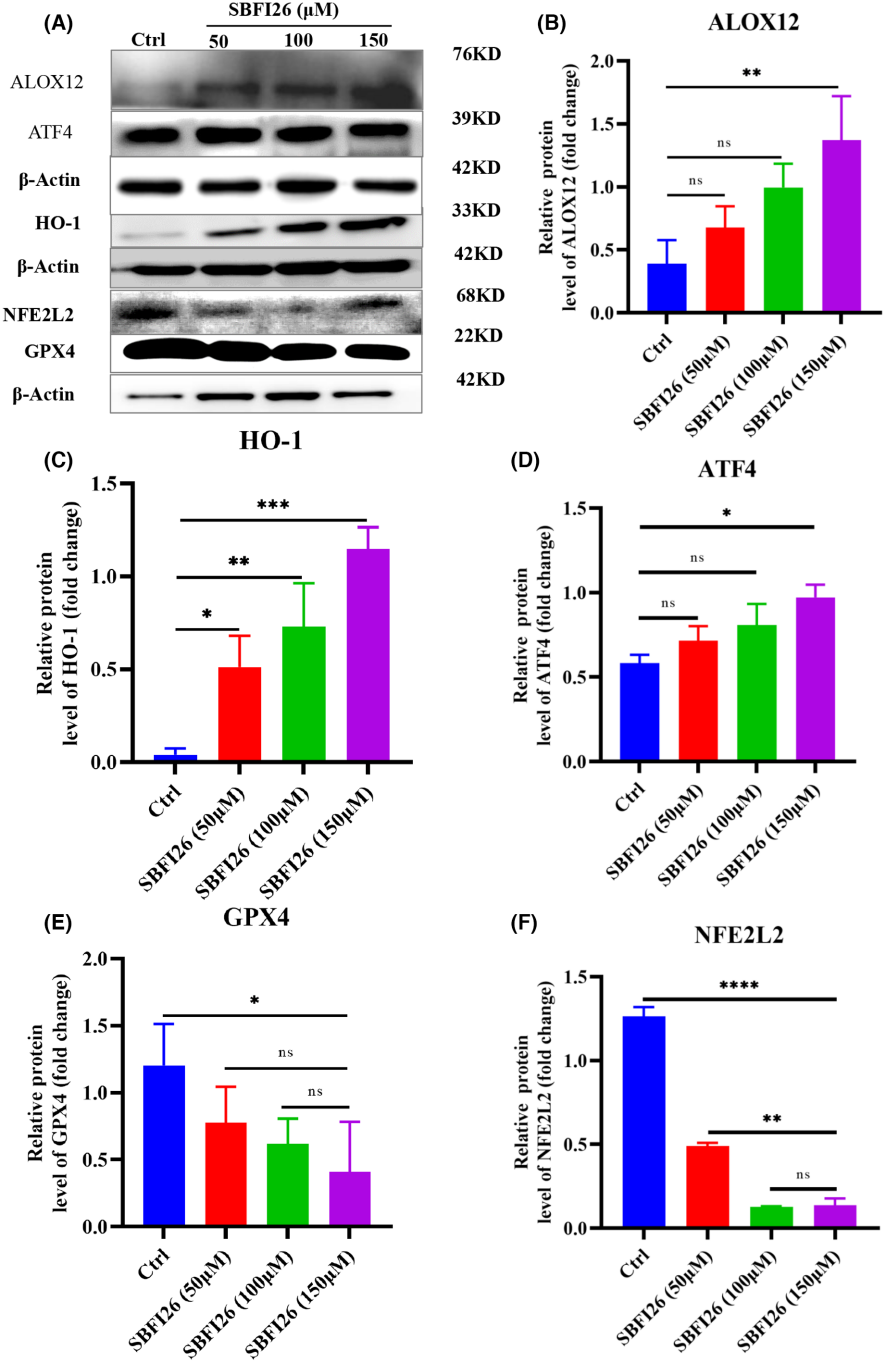
Effects of SBFI26 on protein expressions in MDA‐MB‐231 cells, β‐Actin as internal reference. (A) Western blot analysis of the indicated proteins(ALOX12, HO‐1, NFE2L2, ATF4, GPX4, β‐Actin) following treatment with SBFI26 (50,100,150 μM) for 24 h. Quantitative analyses of ALOX12 (B), HO‐1(C), ATF4 (D), GPX4 (E) and NFE2L2 (F) protein expression levels in MDA‐MB‐231 cells. Data were presented as Mean ± SD. Data were analysed using one‐way ANOVA, with* *p* < 0.05, ** *p* < 0.01, *** *p* < 0.001, **** *p* < 0.0001; ns, not significant.

Gene and protein expression was compared between the SBFI26 treatment and control group by qRT‐PCR and Western blot. Eight DEGs, including ATF3, ATF4, HMOX1 (HO‐1), ALOXE3, SAT1, ALOX15, CHAC1 and TP53, were selected for further qRT‐PCR analysis (Figure 7). The qRT‐PCR results demonstrated that SBFI26 treatment significantly up‐regulated the expression of HMOX1, SAT1, ALOX genes (ALOX5, ALOX12, ALOXE3), CHAC1 and ATF3 genes while down‐regulating NFE2L2 gene expression compared to the control group. Only the 150 μM treatment group showed significant upregulation of TP53, GPX4 and ATF4 gene expression (*p* < 0.05). Notably, except for ALOX15, the expression patterns of these seven DEGs were consistent with the RNA‐seq data. ALOX12 and HMOX1 (HO‐1) exhibited a significantly increased protein level in the SBFI26 group compared to the control group, while ATF4 showed an increasing trend in protein level (Figure 8B–D). On the other hand, NFE2L2 and GPX4 demonstrated a significantly decreased protein level in the SBFI26 group compared to the control group (Figure 8E,F). Although GPX4 exhibited a declining trend at the protein level, its up‐regulation at the gene level suggests potential involvement in protein degradation and non‐functionality, thereby indicating a feedback mechanism that up‐regulates GPX4 gene expression.

### 3.8 Relationship between mRNA levels of ferroptosis genes and clinicopathological parameters in breast cancer patients

Using the GEPIA dataset (Gene Expression Profiling Interactive Analysis) (http://gepia.cancer‐pku.cn/), We conducted a comparative analysis of the mRNA expression levels of SAT1, ALOX5, ATF3, ATF4, HO‐1 and NFE2L2 in breast cancer tissues and normal breast tissues. The results revealed that breast cancer tissues exhibited lower expression levels of SAT1, ALOX5, ALOX12, ATF3, ATF4, GPX4 and NFE2L2 compared to normal tissues; conversely, higher expression levels of ALOX15, ALOXE3 and HO‐1 were observed in breast cancer tissues than in normal tissues (Figure S3A,B). We analysed the expression levels of SAT1, ALOXS, ATF3, ATF4, HO‐1, GPX4 and NFE2L2 in breast cancer tumour stages. The ALOX5, ALOX12, ALOXE3 and ATF3 genes exhibited significant differences, while no significant differences were observed in the genes SAT1, ALOX15, ATF4, HO‐1, GPX4 and NFE2L2 (Figure S3C).

The expression of critical genes associated with ferroptosis strongly correlates with improved prognosis in patients with TNBC. We further investigated the impact of key ferroptosis‐related genes on the survival prognosis of patients with TNBC. In order to explore the correlation between gene expression levels associated with ferroptosis and patient survival, we utilized publicly available datasets (2015 version) and employed the Kaplan–Meier Plotter tool (http://kmplot.com/analysis/index.php?p=service&cancer=breast), following established methodology. The Kaplan–Meier curve and logarithmic ranking test analysis revealed a significant association between ALOX15, ALOX12, ATF4 and GPX4 mRNA levels with overall survival (OS) in all patients with TNBC (*p* < 0.05) (Figure S4). The prognostic significance of ALOX5, ALOXE3, NFE2L2, SAT1, ATF3 and HO‐1 in TNBC exhibits no correlation.

## 4 DISCUSSION

SBFI26 inhibited the proliferation of breast cancer cells (MCF‐7, MDA‐MB‐468, MDA‐MB‐231) in a dose‐dependent and time‐dependent manner. In particular, SBFI26 has a better inhibitory effect on TNBC (MDA‐MB‐231) cells with a high expression of FABP5.^16^, ^19^ This result is consistent with the experimental results of Ke et al. that SBFI26 has an excellent inhibitory effect on highly malignant prostate cancer cells with a high expression of FABP5.^10^, ^23^ These results indicate that SBFI26 can inhibit FABP5 to transport fatty acids and disrupt the process of lipid metabolism, thereby inhibiting cell proliferation and inducing cell death.

The results of GO and KEGG analysis of transcriptome data showed that SBFI26 inhibited MDA‐MB‐231 cell proliferation and induced cell death involving ferroptosis. Ferroptosis is an iron‐dependent, nonapoptotic mode of cell death characterized by lipid ROS accumulation.^56^, ^57^ By comparing the effects of Fer‐1, an iron death inhibitor and Erastin—an inducer, on SBFI26‐induced iron death, we determined the changing trend of biochemical factors closely related to ferroptosis in cells, including MDA, Fe^2+^, Fe, T‐SOD, T‐GSH, GSSG and GSH. The results showed that SBFI26 could induce ferroptosis in TNBC cells with high FABP5 expression.

According to Stockwell, ferroptosis consists of three essential hallmarks: (1) the inactivation of GPX4; (2) excess active iron; (3) polyunsaturated fatty acids (PUFA) of membrane lipids are oxidized.^50^, ^58^, ^59^ Four mechanisms of initiating ferroptosis have been identified. The first category of ferroptosis inducers triggers this process by depleting GSH; the second category directly targets GPX4 inactivation to induce ferroptosis; the third category induces ferroptosis by impairing both GPX4 and CoQ10 through the SQS‐me‐mevalonate pathway, while the fourth category promotes lipid peroxidation by increasing LIP (Labile iron pool) or iron oxide.^38^, ^41^, ^42^ Therefore, iron, lipids, and ROS constitute the fundamental components underlying ferroptosis.^60^, ^61^

Iron is necessary for lipid peroxide accumulation and ferroptosis.^62^ Therefore, iron uptake, transport and storage will have a particular regulatory effect on ferroptosis. In this study, after SBFI26 treatment, Fe^2+^ and total Fe content increased significantly with dose and incubation time. Organisms tightly regulate the balance of iron within their systems to maintain homeostasis. Excess‐free iron can react with hydrogen peroxide (H_2_O_2_) through the Fenton reaction to form hydroxyl radicals and highly reactive ROS to attack cell membranes. When intracellular iron homeostasis is imbalanced, resulting in increased divalent iron ions within the cell, there is a corresponding increase in the production of toxic ROS substances mediated by iron ions, ultimately leading to ferroptosis.^63^, ^64^

Heme oxygenase‐1 (HO‐1) catalyses the catabolism of heme to generate ferrous ions, which serve as a crucial source of intracellular iron ions.^65^ After treatment with SBFI26, both qPCR and Western blot analysis revealed a significant increase in mRNA and protein expression levels of HO‐1 and its upstream regulatory gene ATF4, accompanied by a substantial elevation in total cellular iron and ferrous ion concentrations.^66^, ^67^ On the other hand, ATF4‐mediated transcriptional expression of GSH‐degrading enzyme CHAC1 enhanced cystine starvation‐induced ferroptosis.^60^ our result indicated GSH was significantly reduced after SBFI26 treatment.

Iron‐dependent lipid ROS accumulation was implicated in ferroptosis across all pathways.^44^, ^57^ Lipid metabolism was intricately linked to ferroptosis, with PUFAs being highly susceptible to lipid peroxidation and serving as essential components for the execution of ferroptotic cell death.^56^ The transport of fatty acids by FABP5 and FABP7 in TNBC cells was impeded by SBFI26, resulting in perturbations in fatty acid metabolism. The disturbance of intracellular lipid metabolism is consistent with the critical biological characteristics of ferroptosis. ALOXs are non‐heme iron‐containing dioxygenases that catalyse the peroxidation and esterification of PUFAs, producing various biologically active lipid intermediates, including MDA.^49^, ^65^, ^68^, ^69^

P53 plays a crucial role in ferroptosis,^42^, ^70^, ^71^ induces SAT1 expression, promoting ALOX15 function to enhance cell ferroptosis.^72^, ^73^ Wei Gu et al. demonstrated that p53‐ALOX12 can promote GSH‐independent ferroptosis.^74^ The lipid oxidase ALOX12 was identified as a critical regulator of p53‐dependent ferroptosis by free oxidation of polyunsaturated fatty acid chains in cell membrane phospholipids leading to cellular ferroptosis. Similarly, ALOXE3 was also found to induce ferroptosis like ALOX12.^74^ Following treatment with SBFI26, mRNA expression levels of TP53, SAT1, ALOX15, ALOX12, ALOX5 and ALOXE3 genes were significantly upregulated. Concurrently, intracellular MDA content significantly increased while T‐SOD levels significantly decreased. Our results suggest that SBFI26 may induce ferroptosis through the TP53‐SAT1‐ALOX signalling pathway.

Glutathione peroxidase 4 (GPX4) plays an essential role in ferroptosis involving ATF3,^75^, ^76^ NFE2L2 and a membrane‐embedded XC system consisting of a dimer of SLC3A2 and SLC7A11. ATF3 suppressed SLC7A11 expression and promoted ferroptosis. NFE2L2, also known as NRF2, plays a crucial role in regulating the antioxidant responses of cells.^77^ The level of NFE2L2 has been directly correlated with ferroptosis sensitivity, as increased expression of NFE2L2 prevents ferroptosis, whereas decreased NFE2L2 enhances the sensitivity of cancer cells to pro‐ferroptosis agents.NFE2L2 can enhance the ability of the Xc‐ system to protect cells from ferroptosis, and cystine uptake mediated by the Xc‐ system is essential for the synthesis of GSH.^78^ Our results showed that ATF3 gene expression was up‐regulated and NFE2L2 expression was down‐regulated at both gene and protein levels. Our study showed that the up‐regulation of GPX4 mRNA expression was consistent with the transcriptome results, but its protein level was down‐regulated by western blot. This may be related to the up‐regulation of CHAC1 expression and down‐regulation of NFE2L2 expression, which reduces intracellular GSH levels and impairs GPX function.

The analysis of the GEPIA dataset revealed that ALOX15, ALOXE3, and HO‐1 exhibited higher expression levels, while ALOX12 showed lower expression levels in breast cancer tissues compared to normal tissues. Significant differences were observed in breast cancer tumour staging for ALOX5, ALOXE3, ALOX12, and ATF3 genes. However, no significant differences were found for SAT1, ALOX15, ATF4, HO‐1, GPX4 and NFE2L2 Genes. Furthermore, the expression of these critical genes related to ferroptosis was closely associated with improved prognosis in patients with TNBC. The treatment of SBFI26 significantly altered the expression of these genes in MDA‐MB‐231 cells and further analysis using the GEPIA dataset confirmed that SBFI26 could induce ferroptosis by regulating the critical genes involved in this process. Analysis of Kaplan–Meier curves and logarithmic sequencing tests revealed that mRNA levels of ALOX15, ALOX12, ATF4, and GPX4 were significantly associated with overall survival (OS) in all TNBC patients (*p* < 0.05). However, there was no correlation between the prognostic value of ALOX5, ALOXE3, NFE2L2, SAT1, ATF3 and HO‐1 in TNBC. The results obtained from this research indicate that targeting ALOX15 could be a potential avenue for treating breast cancer. Following SBFI26 intervention, the real‐time PCR results demonstrated significant up‐regulation of ALOX15 and its upstream genes SAT1 and TP53, promoting cellular ferroptosis.

Our work demonstrates that SBFI26 can disrupt the balance of lipid metabolism by disrupting fatty acid transport and ultimately promote ferroptosis through lipid peroxidation. However, the subject of further study will be how SBFI26 enhances the expression of TP53 and HMOX1.

## 5 CONCLUSIONS

**FIGURE 9:**
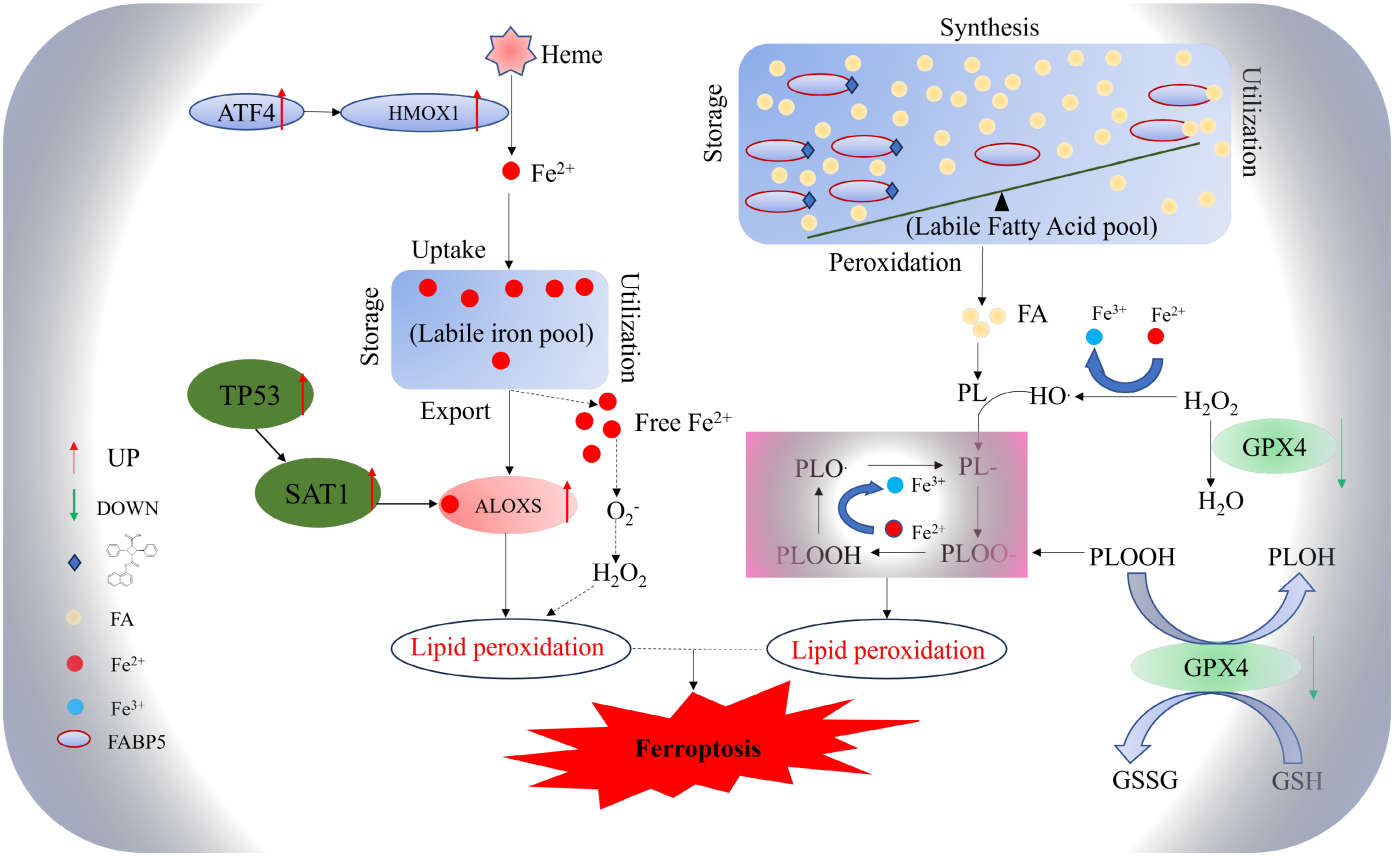
Mechanism of SBFI26‐induced ferroptosis in triple‐negative breast cancer MDA‐MB‐231 cells.

Through transcriptome analysis, we have identified that SBFI26 drives cellular ferroptosis by triggering a ferrous ion‐mediated Fenton reaction, promoting intracellular lipid peroxidation by activating the ALOXS family and causing biofilm stress damage. Additionally, it decreases GPX4 antioxidant system function and increases sensitivity to cellular ferroptosis via these pathways. As shown in Figure 9, SBFI26 disrupts the balance of the fatty acid pool by inhibiting FABP5's function in transporting fatty acids, leading to lipid peroxidation and ultimately inducing ferroptosis in TNBC cells. The involved pathways or pathway nodes include “ATF4‐HO1‐Fe^2+^”, “TP53‐SAT1‐ALOX15/ ALOXE3‐Lipid ROS”, “ATF3/NFE2L2‐Xc system‐GPX4” and “CHAC1‐GSH‐GPX4”.

## AUTHOR CONTRIBUTIONS

**Gang He:** Conceptualization (lead); data curation (lead); formal analysis (lead); funding acquisition (lead); investigation (lead); methodology (lead); project administration (lead); resources (lead); software (lead); supervision (lead); validation (lead); visualization (lead); writing – original draft (equal); writing – review and editing (lead). **Yiyuan Zhang:** Data curation (lead); formal analysis (lead); investigation (lead); methodology (lead); resources (equal); software (lead); validation (lead); visualization (equal); writing – original draft (equal); writing – review and editing (equal). **Yanjiao Feng:** Formal analysis (equal); investigation (equal); methodology (equal); resources (equal); software (equal); validation (equal); visualization (equal); writing – review and editing (equal). **Tangcong Chen:** Data curation (equal); formal analysis (equal); investigation (equal); methodology (equal); resources (equal); software (equal); validation (equal); visualization (equal); writing – review and editing (equal). **Mei Liu:** Conceptualization (equal); data curation (equal); formal analysis (equal); investigation (equal); methodology (equal); resources (equal); software (equal); validation (equal); writing – review and editing (equal). **Yue Zeng:** Data curation (equal); formal analysis (equal); investigation (equal); methodology (equal); resources (equal); software (equal); validation (equal); visualization (equal); writing – review and editing (equal). **Xiaojing Yin:** Data curation (equal); formal analysis (equal); investigation (equal); methodology (equal); resources (equal); software (equal); validation (equal); visualization (equal); writing – review and editing (equal). **Shaokui Qu:** Data curation (equal); formal analysis (equal); investigation (equal); methodology (equal); resources (equal); software (equal); validation (equal); visualization (equal); writing – review and editing (equal). **Lifen Huang:** Data curation (equal); formal analysis (equal); methodology (equal); resources (equal); software (equal); validation (equal); visualization (equal); writing – review and editing (equal). **Youqiang Ke:** Conceptualization (equal); investigation (equal); software (equal); supervision (equal); validation (equal); writing – review and editing (equal). **Li Liang:** Data curation (equal); formal analysis (equal); investigation (equal); resources (equal); validation (equal); writing – review and editing (equal). **Jun Yan:** Conceptualization (equal); formal analysis (equal); investigation (equal); resources (equal); software (equal); writing – review and editing (equal). **Wei Liu:** Conceptualization (equal); investigation (equal); supervision (lead); validation (equal); visualization (equal); writing – review and editing (equal).

## FUNDING INFORMATION

This research was supported by the Sichuan Science and Technology Support Program, Grant Numbers: 2019YFH0054 and 2020YFH0205, and the Personnel Training Quality and Teaching Reform Project of Chengdu University, Grant Number: cdjgb2022156.

## CONFLICT OF INTEREST STATEMENT

The authors declare no conflicts of interest.

## Supporting information


Figure S1.

Figure S2.

Figure S3.

Figure S4.

## Data Availability

The NCBI public database may be accessed at the following site. The raw readings were submitted there https://www.ncbi.nlm.nih.gov/sra/PRJNA1001366.
