# Supplementary figures and images for: SBFI26 induces triple‐negative breast cancer cells ferroptosis via lipid peroxidation
Source: J Cell Mol Med. 2024 Mar 22;28(7):e18212. doi: 10.1111/jcmm.18212 (PMC10958404; doi:10.1111/jcmm.18212)

## Slide 1
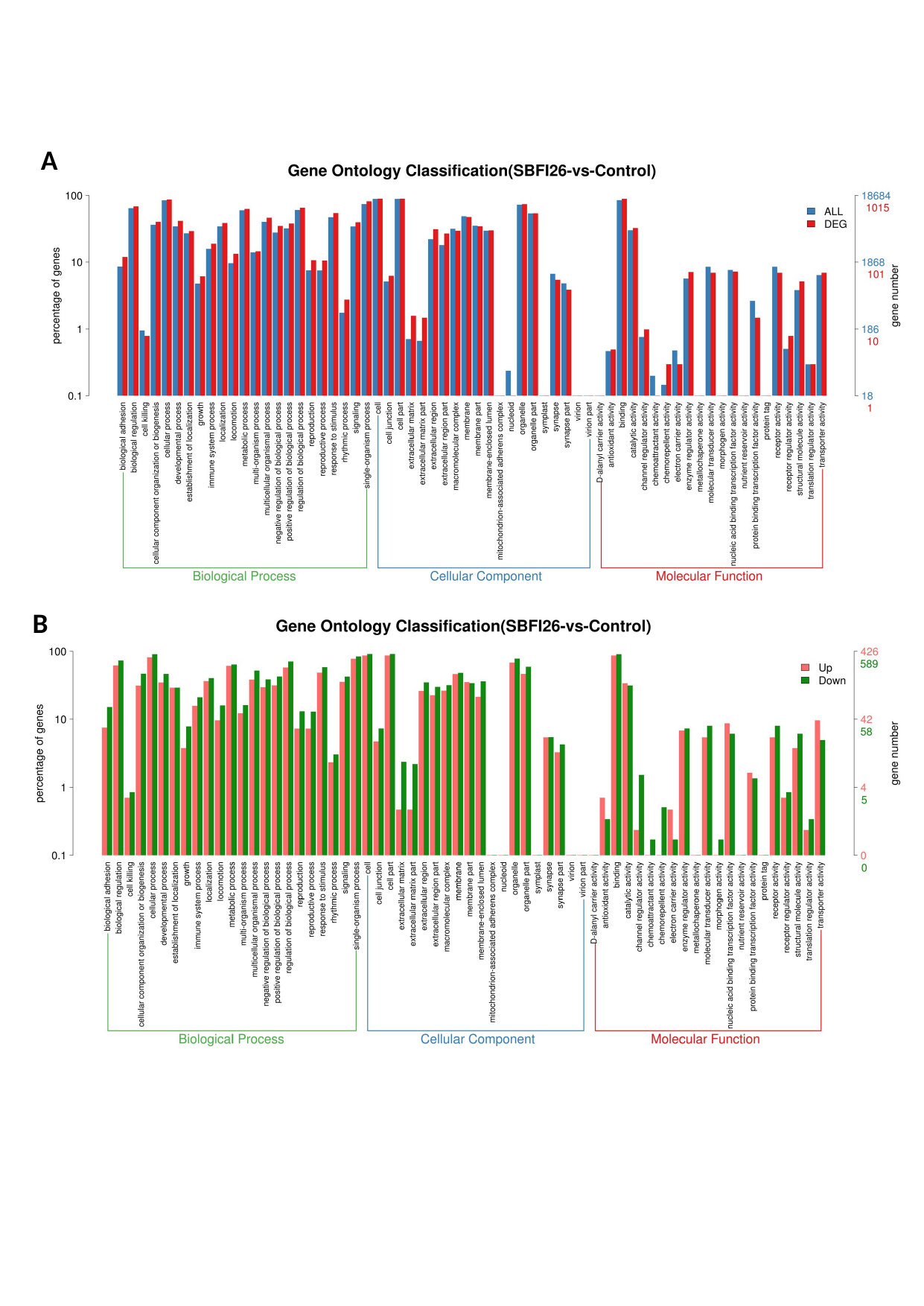

A
B

Supplement: Supplementary file 1 — Figure S1. [file JCMM-28-e18212-s002.pptx]

## Slide 1
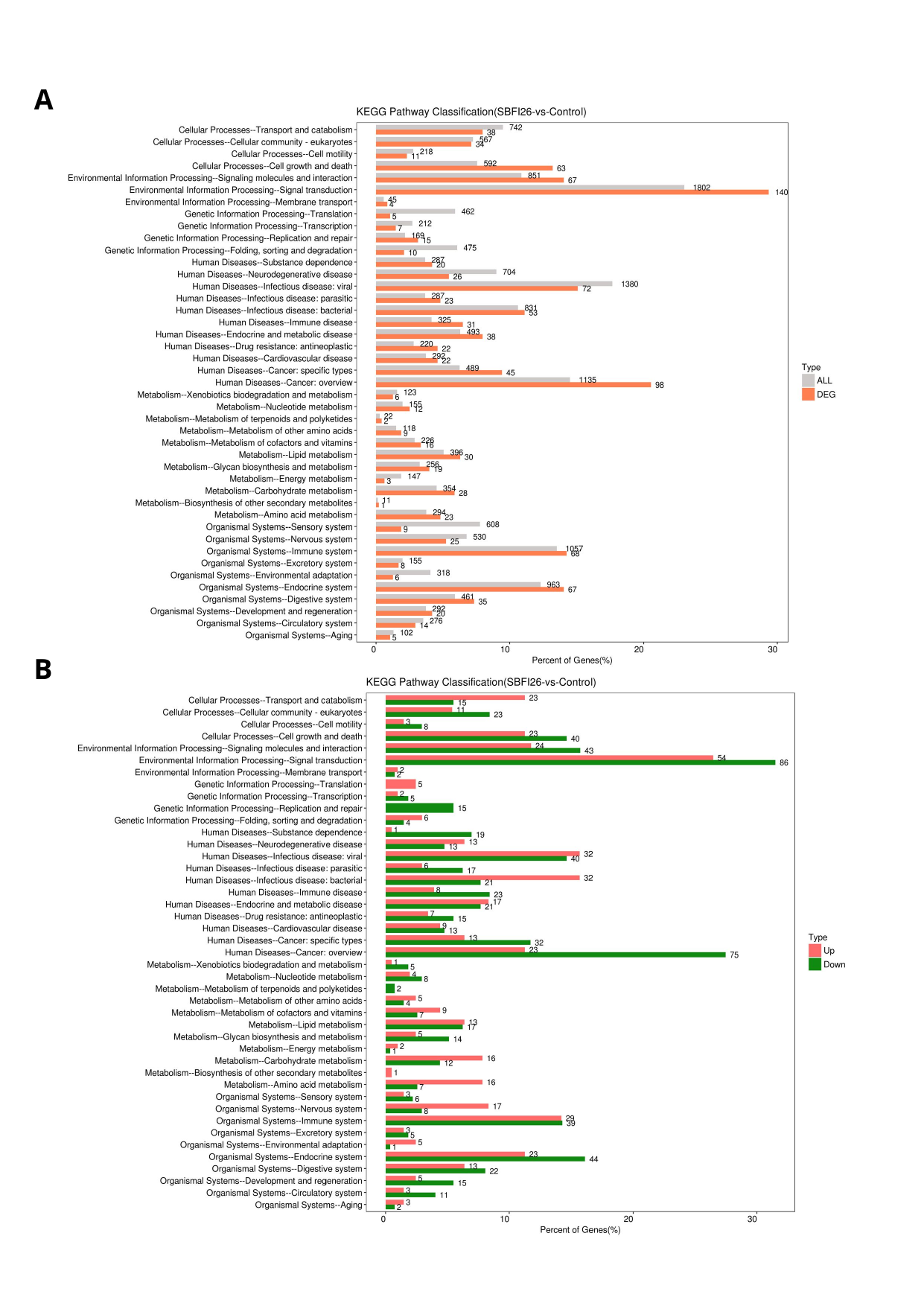

A
B

Supplement: Supplementary file 2 — Figure S2. [file JCMM-28-e18212-s004.pptx]

## Slide 1
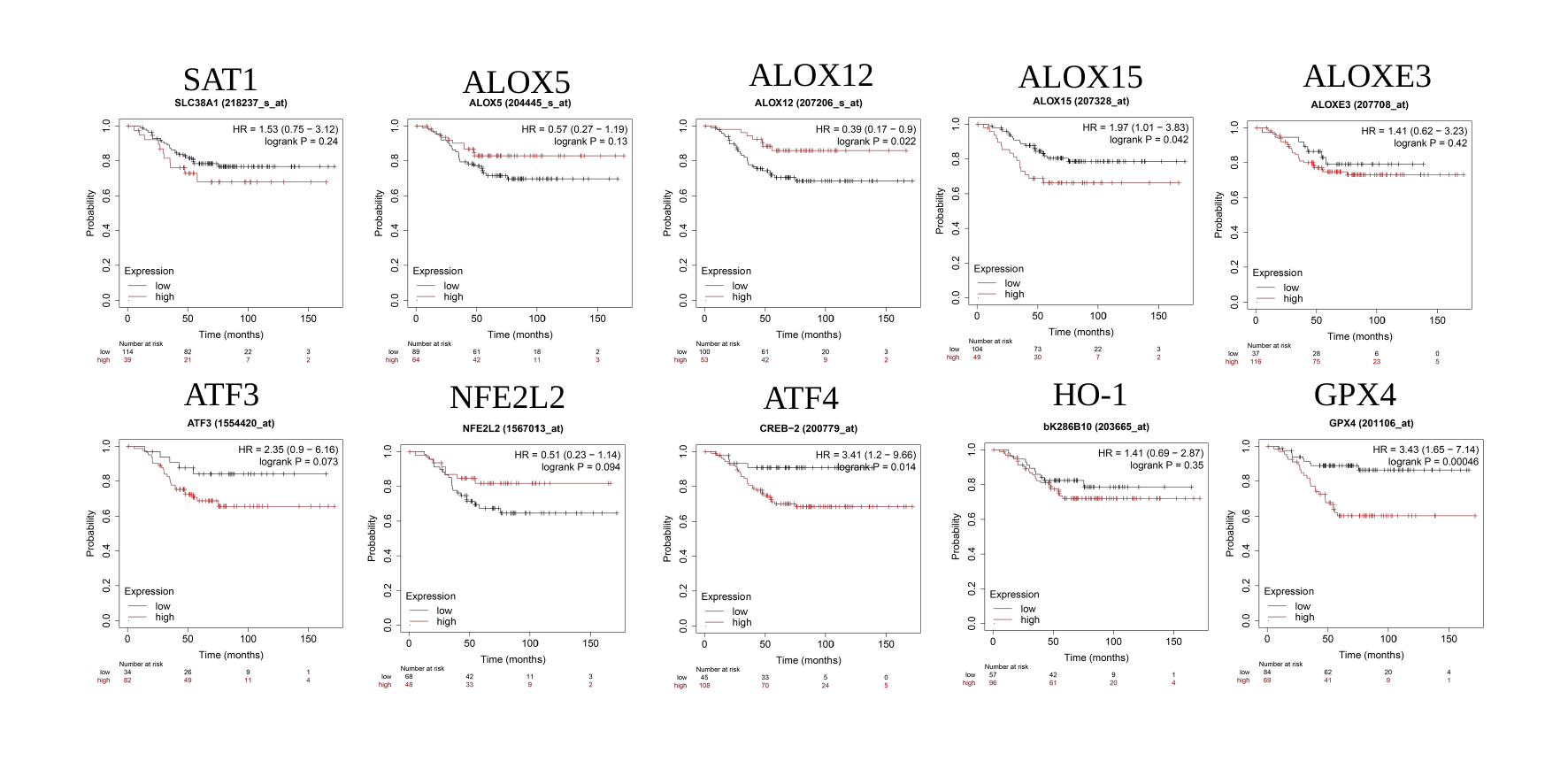

ALOX12
ALOXE3
ALOX15
SAT1
ALOX5
ATF3
HO-1
GPX4
NFE2L2
ATF4

Supplement: Supplementary file 4 — Figure S4. [file JCMM-28-e18212-s003.pptx]
