# Supplementary material for: SBFI26 induces triple‐negative breast cancer cells ferroptosis via lipid peroxidation
Source: J Cell Mol Med. 2024 Mar 22;28(7):e18212. doi: 10.1111/jcmm.18212 (PMC10958404; doi:10.1111/jcmm.18212)
Supplement: Supplementary file 3 — Figure S3. [file JCMM-28-e18212-s001.pptx]

## Slide 1
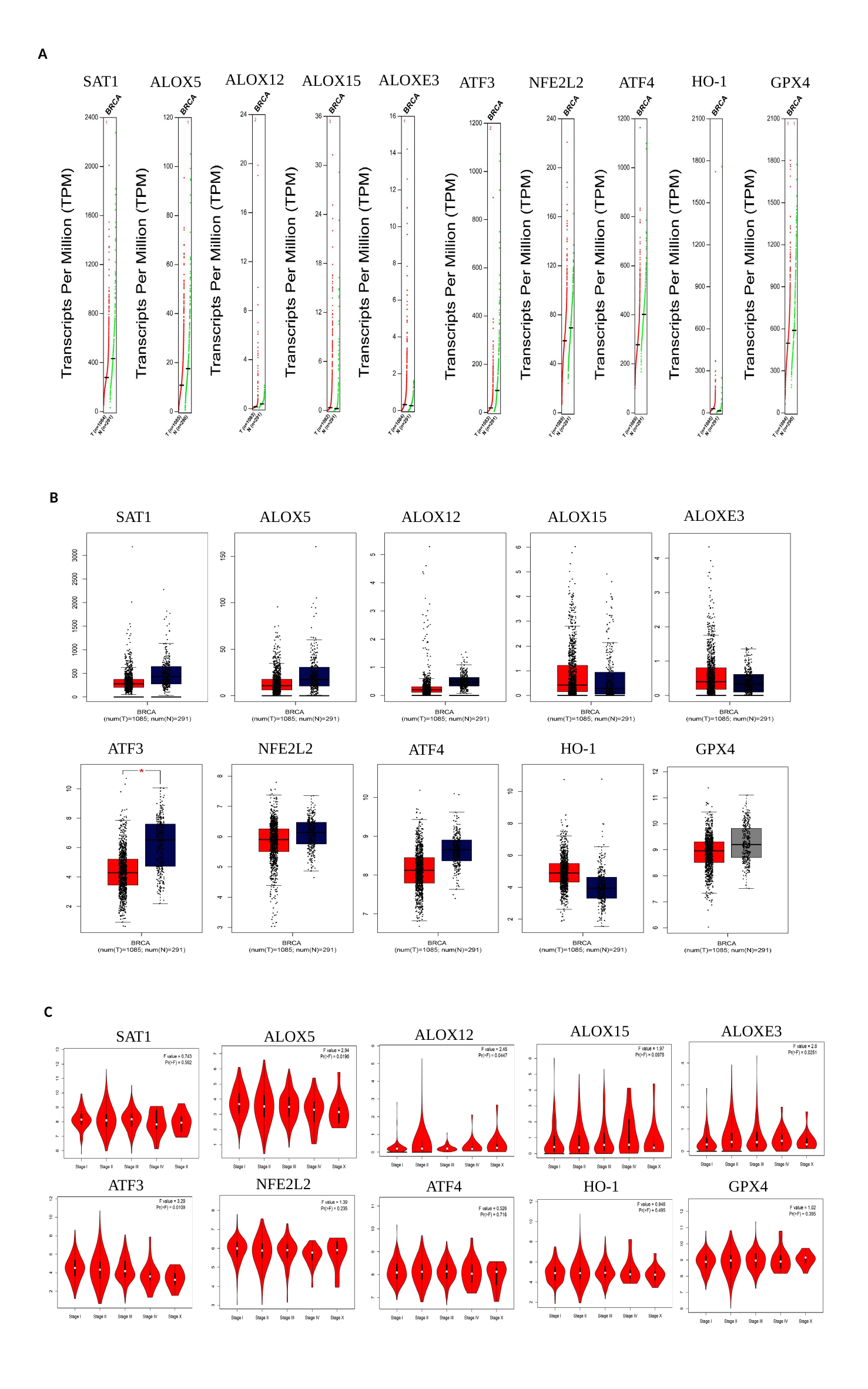

A
ALOX12
ALOXE3
ALOX15
SAT1
ALOX5
HO-1
ATF3
NFE2L2
ATF4
GPX4
B
ALOXE3
SAT1
ALOX5
ALOX12
ALOX15
ATF3
NFE2L2
HO-1
GPX4
ATF4
C
ALOX15
ALOXE3
ALOX12
ALOX5
SAT1
NFE2L2
ATF3
ATF4
GPX4
HO-1
